# Implication of atrial size and function in adults with repaired Tetralogy of Fallot and the effect of treatment by ramipril: the APPROPRIATE randomised trial

**DOI:** 10.1186/1532-429X-15-S1-E93

**Published:** 2013-01-30

**Authors:** Beatrice Bonello, Anselm Uebing, Wei Li, Gerhard Diller, Michael Gatzoulis, Sonya V Babu-Narayan

**Affiliations:** 1 Congenital Heart Diseases, CHU Timone enfants, Marseille, France; 2 CMR Unit, Royal Brompton Hospital, London, UK; 3 Adults Congenital Heart Disease, Royal Brompton Hospital, London, UK; 4 Imperial College, Londoon, UK

## Background

Background: Patients with repaired tetralogy of Fallot (rtoF) are at risk of supra-ventricular tachycardia due to atrial dilatation. To date, the impact of atrial size and function has been poorly studied in this population. Angiotensin-converting enzyme (ACE ) inhibitor are known for their beneficial effects on atrial and ventricular remodelling in patients with cardiomyopathy. A previous study (APPROPRIATE study) has demonstrated their beneficial effect on ventricular longitudinal contraction in patients with rtoF. We aimed to study first the impact atria volume and function and second, the effect of ACE inhibitor ramipril in adults with rtoF.

## Methods

Methods: We studied prospectively 61 patients with rtoF from the APPROPRIATE study cohort (median age 29.5 [interquartile range 22-38] years). A double-blinded, placebo-controlled study of 6 months of ramipril was performed. All patients underwent cardiac magnetic resonance imaging (CMR) with both atria and ventricle volume and function measurements, echocardiography, neurohormonal analysis, and objective cardiopulmonary testing at baseline and follow-up. Values are expressed as median [interquartile range].

## Results

Results: At baseline, all patients were in sinus rhythm. Right atrium (RA) maximal area index was 13.7 [12.2-15.2] cm^2^/m^2^. Right ventricle (RV) end-diastolic volume index was (129 [109-144] ml/m^2^) and RV ejection fraction was 53 [47-49]. It was correlated with age (r=0.56, p<0.0001), RV end diastolic volume index (r=0.25, p=0.04), BNP (r=0.4, p=0.0007), inversely correlate with right ventricle (RV) ejection fraction (r=-0.26, p=0.04). LA size and volume were normal and not correlated with any parameter, probably because there was no associated left heart disease and all our studied patients had normal left ventricle volume and ejection fraction. After 6 months of treatment, no arrhythmia occurred. In the ramipril group, LA maximal volume decreased (p=0.01), anterior septum excursion movement increased, BNP level decreased (p=0.0004) compared to the placebo group.

## Conclusions

Conclusions: RA size was a marker of RV dilatation and systolic dysfunction. Ramipril appears to have beneficial action on left heart with atrial remodelling in patients with rtoF. However ramipril had no effect on the right heart. Further studies with longer follow-up and larger group are needed.

## Funding

British Heart Foundation Fellowship (SVB-N).

French Federation of Cardiology (BB).

Unrestricted Actelion educational grant (GD).

The study was supported by the NIHR Cardiovascular Biomedical Research Unit of Royal Brompton and Harefield NHS Foundation Trust and Imperial College London.

